# Usefulness of Bidirectional Barbed Sutures for Temporary Closure of Entry Hole for Delta‐Shaped Anastomosis in Minimally Invasive Gastrectomy

**DOI:** 10.1111/ases.70046

**Published:** 2025-03-11

**Authors:** Hironori Tsujimoto, Hiroyuki Horiguchi, Yoshihisa Yaguchi, Naoyuki Uehata, Risa Kariya, Asuma Ide, Keita Kouzu, Hideki Ueno

**Affiliations:** ^1^ Department of Surgery National Defense Medical College Tokorozawa Japan

**Keywords:** delta‐shaped anastomosis, entry hole, robotic surgery

## Abstract

**Introduction:**

Laparoscopic and robotic gastrectomies have become standard procedures for the treatment of gastric cancer. Among the reconstruction methods used following distal gastrectomy, the Billroth‐I technique is often preferred owing to its low complication rates. Delta‐shaped anastomosis, a method that eliminates the need for a mini‐laparotomy, represents a significant advancement in minimally invasive surgeries. In this report, we aim to present a novel technique using bidirectional barbed sutures for temporary closure of the entry hole during delta‐shaped anastomosis in laparoscopic and robotic gastrectomies.

**Materials and Surgical Technique:**

The entry hole was closed using a bidirectional barbed suture, starting centrally to prevent overlapping of the gastric and duodenal staple lines. The suture length was meticulously adjusted to align with the stapler dimensions. All the procedures were successfully completed without any complications in both laparoscopic and robotic gastrectomies. Bidirectional barbed sutures enabled precise tissue alignment and prevented slippage, thereby facilitating secure, full‐thickness closure of the entry hole while minimizing the risk of incomplete stapler firing.

**Conclusion:**

Bidirectional barbed sutures offer a safe and feasible alternative option for the temporary closure of the entry hole during a stapled anastomotic technique in robotic and laparoscopic gastrectomies.

## 1 Introduction

As Kitano et al. first reported laparoscopy‐assisted gastrectomy in 1991 [1], it has become the standard treatment for both early and advanced gastric cancers [2]. Reconstruction following distal gastrectomy typically involves the Billroth‐I, Billroth‐II, or Roux‐en‐Y methods, each having its unique advantages and disadvantages [3, 4, 5, 6]. Among these, Billroth‐I is the simplest method and offers the additional benefit of avoiding complications such as Petersen's hernia or Roux‐en‐Y stasis syndrome [7, 8].

The delta‐shaped anastomosis technique, introduced by Kanaya et al. in 2002, is a groundbreaking approach. It only requires a linear stapler that is inserted through a 12‐mm port, eliminating the need for a mini‐laparotomy to insert a circular stapler [9]. In our previous study, we demonstrated that total laparoscopic gastrectomy with complete intracorporeal anastomosis provided superior postoperative pain management in elderly patients with gastric cancer compared to laparoscopic gastrectomy using a circular stapler with mini‐laparotomy [10]. Consequently, Billroth‐I reconstruction with delta‐shaped anastomosis using a linear stapler may offer additional benefits such as improved esthetics, better pain control, and fewer complications than Billroth‐II or Roux‐en‐Y reconstruction.

Barbed sutures are widely utilized in laparoscopic and robotic surgeries owing to their unique design. Barbs allow the suture to advance unidirectionally, thereby preventing loosening. Bidirectional barbed sutures are specially designed with barbs oriented in opposite directions from the midpoint of the suture and needles attached at both ends. This configuration enhances the strength and is primarily used to close the vaginal cuff in laparoscopic gynecological procedures [11, 12].

In this report, we aim to present a novel surgical technique using bidirectional barbed sutures for temporary closure of the entry hole during delta‐shaped anastomosis in minimally invasive gastrectomy. This method enhances stapler application and may contribute to optimizing the procedure.

## 2 Ethics Approval and Consent to Participate

All protocols were approved by the Institutional Review Board of the National Defense Medical College (Permission number: 5119). Written consent was obtained prior to the study.

## 3 Materials and Surgical Technique

Port placement was performed under general anesthesia as previously described [13]. Lymph node dissection was performed based on the tumor stage. The duodenum and stomach were resected using either a blue cartridge 45‐mm linear stapler for robotic surgery (ENDOWRIST Stapler; Intuitive Surgical, Sunnyvale, CA, US) or a blue cartridge 60‐mm linear stapler for laparoscopic surgery (Echelon Flex Powered Endopath; Ethicon, Tokyo, Japan). The resected specimen was extracted through an extended umbilical incision that was sufficiently large for removal. As previously reported, small incisions were made in the duodenum and stomach, followed by a gastroduodenal anastomosis using a linear stapler (blue cartridge).

**FIGURE 1:**
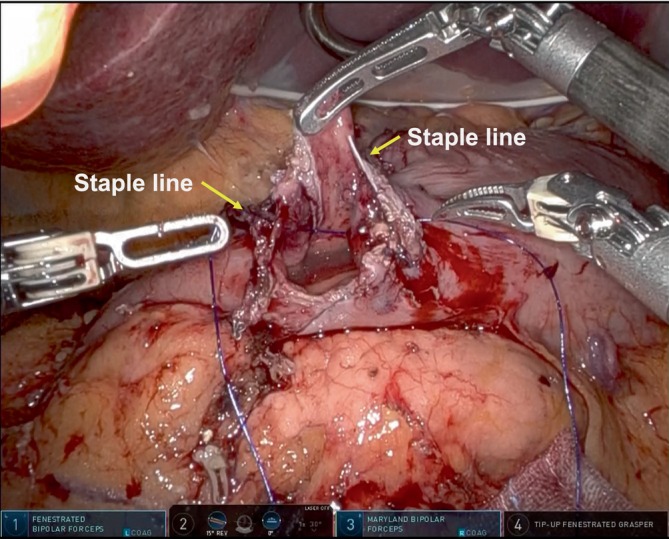
All layers at the center of the gastric and duodenal walls are sutured with a bidirectional barbed suture (STRATAFIX Spiral PDS Plus Bidirectional, Ethicon, Tokyo, Japan), taking care to avoid overlap of the stapler lines of the stomach and the duodenum to ensure adequate blood flow. Allows indicate staple lines of the stomach and duodenum.

**FIGURE 2:**
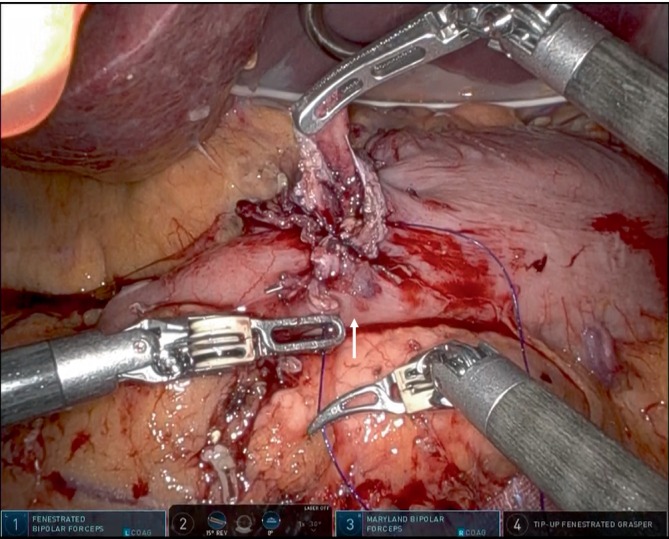
Continuous suturing is then performed from the center toward the lesser curvature, ensuring that all layers are included. The sutured tissue is then drawn from the greater curvature side toward the center. Arrow indicates the direction of tissue pull toward the center with the first arm.

Subsequently, the entry hole was temporally closed. Initially, all layers at the center of the gastric and duodenal walls were sutured with a bidirectional barbed suture (STRATAFIX Spiral PDS Plus Bidirectional; Ethicon), while avoiding overlap with the stapler lines to ensure adequate blood flow (Figure 1). Continuous suturing was then performed from the center toward the greater curvature to ensure the inclusion of all layers. The sutured tissue was subsequently drawn from the greater curvature toward the center (Figure 2). At last, continuous suturing was performed from the center to the side of the lesser curvature, followed by drawing the tissue from the lesser curvature toward the center. This maneuver was carefully adjusted so that the final anastomotic diameter was the ideal size for the surgeon's preference.

**FIGURE 3:**
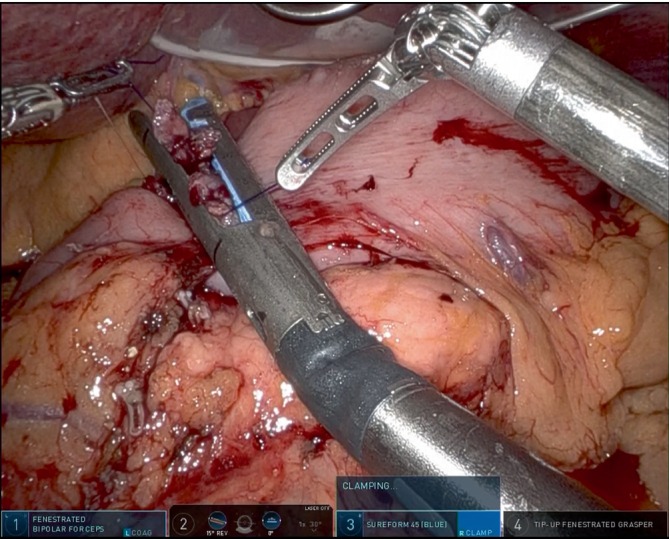
The suture length was able to be meticulously adjusted to align with the stapler dimensions. The suture on the greater curvature side is managed using forceps on the fourth arm, while the suture on the lesser curvature side is controlled by forceps on the 1st arm. The final closure is completed with a 45 mm‐linear stapler (blue cartridge) through the third arm. This suturing technique does not require the assistance of an assistant.

In robotic surgery, the suture threads on the greater and lesser curvatures were pulled in the appropriate direction using forceps on the fourth arm and first arm, respectively (Figure 3). The final (permanent) closure (twice if necessary) was completed with a 45‐mm linear stapler (blue cartridge) using the third arm. This suturing technique does not require the assistance of an assistant.

**FIGURE 4:**
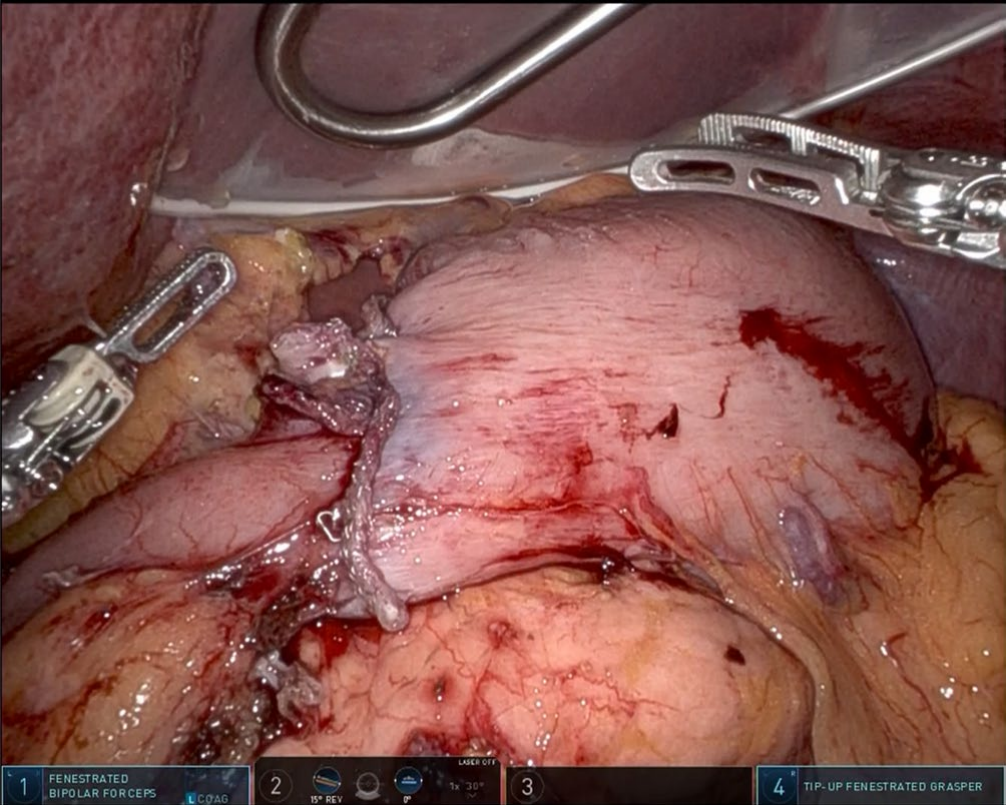
Photograph taken after the completion of delta‐shaped anastomosis reconstruction performed during robotic distal gastrectomy.

A leak test was conducted to ensure anastomotic integrity; a Blake drain (19 Fr J‐VAC drain; Covidien, Dublin, Ireland) is placed before completing the surgery (Figure 4). We have applied this technique to two robotic cases and one laparoscopic case without any difficulty, and all patients were discharged according to the clinical pathway without stenosis or other complications.

## 4 Discussion

The use of bidirectional barbed sutures for temporary closure of the entry hole during delta‐shaped anastomoses is highly effective. A key advantage of this technique is that initiating closure from the center prevents overlapping of staple lines on the gastric and duodenal sides, eliminating the need for additional ligation procedures. Furthermore, the barbed suture facilitates precise length adjustment from both the lesser and greater curvature sides, enabling the temporary closure length to be tailored to the stapler size (45 or 60 mm). This full‐thickness closure prevents tissue slippage and ensures complete suturing during stapler application. However, if there is concern about stenosis with a single staple, multiple staples should be used.

From a cost perspective, temporary closure has traditionally been performed using 3‐0 Vicryl sutures (Ethicon) at the edge of the lesser curvature, center, and edge of the greater curvature, which cost approximately 1500 JPY in Japan. The bidirectional barbed sutures cost approximately 5000 JPY. Okabe et al. used a stapler for inguinal hernia entry hole closure, which simplifies the procedure but costs approximately 38 000 JPY [14]. Hosoda et al. and Kikuchi et al. reported using a three‐point intracorporeal interrupted hand‐sewn technique for temporary closure in laparoscopic and robotic surgeries [15, 16]. However, in standard laparoscopic and robotic procedures, three forceps are available to pull the suture. For larger entry holes or fragile duodenal walls, more than four sutures may be required. In such cases, three‐point sutures may lead to tissue slipping, preventing suturing of all layers when firing with the stapler. Even in such cases, the tissue can be pulled to the center using a bidirectional barbed suture and stapled at the desired length.

Recently, functional end‐to‐end and delta‐shaped intracorporeal anastomoses have been reported not only in gastric cancer but also in colorectal cancer [17, 18]. Owing to their versatility and effectiveness, the use of bidirectional barbed sutures for temporary closure of entry holes may become increasingly popular.

In conclusion, this approach offers several advantages: the staple lines of the stomach and duodenum do not overlap, assistant support is not required, all layers are stapled effectively, and the stapler can be applied at the desired length to fire.

## Author Contributions

Hironori Tsujimoto devised the surgical procedure. Hironori Tsujimoto, Hiroyuki Horiguchi, Yoshihisa Yaguchi, Naoki Uehata, Risa Kariya, Asuma Ide, and Keita Kouzu conceived and designed this procedure. Hironori Tsujimoto prepared the manuscript. Hideki Ueno supervised the study.

## Ethics Statement

The study protocol was approved by the Institutional Review Board of the National Defense Medical College (Permission number: 5119).

## Consent

Informed consent for participation in the study or its equivalents was obtained from all patients.

## Conflicts of Interest

The authors declare no conflicts of interest.

## Data Availability

The data that support the findings of this study are available from the corresponding author upon reasonable request.
